# Incidence and Risk Factors of Complications After Non-operating Room Anesthesia in Children: A Prospective Observational Study

**DOI:** 10.7759/cureus.107655

**Published:** 2026-04-24

**Authors:** Hamza Kassimi, Khalil Abou Elalaa, Hassan Alaoui, Mustapha Bensghir

**Affiliations:** 1 Anesthesiology, Mohammed 5 Military Teaching Hospital, Rabat, MAR

**Keywords:** complications, non-operating room anesthesia, patient safety, pediatric anesthesia, risk factors, sedation

## Abstract

Background

Non-operating room anesthesia (NORA) is increasingly utilized in pediatric practice, but complications remain a concern, particularly in resource-limited settings.

Objective

The objective of this study is to determine the incidence of complications during pediatric NORA and identify associated risk factors.

Methods

This prospective observational study included 300 children undergoing sedation or anesthesia for diagnostic and therapeutic procedures in radiology and endoscopy departments of a university hospital. Demographics, medical history, procedural details, and complications were recorded. Univariable and multivariable logistic regression analyses were performed to identify independent risk factors.

Results

The overall complication rate was 8.7% (26/300). Respiratory complications were most frequent (57.7%), followed by emergence agitation (30.8%). Independent risk factors included respiratory distress (adjusted OR = 6.845; 95% CI: 1.986-23.583; p = 0.002), altered consciousness (aOR = 9.127; 95% CI: 1.124-74.123; p = 0.038), and recent upper respiratory infection (aOR = 4.921; 95% CI: 1.042-23.230; p = 0.044). Preanesthetic consultation was protective (aOR = 0.389; 95% CI: 0.154-0.982; p = 0.046). No mortality occurred.

Conclusions

Complications during pediatric NORA remain significant. Careful patient selection, thorough preanesthetic evaluation, and awareness of risk factors can improve safety. Systematic preanesthetic consultation should be implemented for all pediatric NORA procedures.

## Introduction

Non-operating room anesthesia (NORA) has become an integral component of pediatric medical care, with an increasing number of diagnostic and therapeutic procedures requiring sedation or anesthesia outside the traditional operating room environment [1]. These procedures include magnetic resonance imaging (MRI), computed tomography (CT), endoscopy, bone marrow biopsies, and various interventional radiology procedures. The unique challenges of NORA include unfamiliar environments, equipment limitations, inadequate monitoring facilities, and often limited access to the patient during procedures [2].

Pediatric patients present particular challenges for NORA due to their inability to cooperate, anatomical and physiological differences, and the need for immobility during diagnostic imaging [3,4]. While NORA facilitates essential medical procedures in children, it carries inherent risks that may differ from those encountered in conventional operating room settings. Studies report that in pediatric NORA, the incidence of complications can be as low as 2-3% in well-resourced settings but can rise to 8.6% or more in low- and middle-income countries [5,6].

Despite the growing body of literature on pediatric NORA, there remains a paucity of data from Low-and Middle-Income Countries, where healthcare systems face unique constraints. Furthermore, few studies have comprehensively examined the protective role of preanesthetic evaluation in reducing complications during pediatric NORA procedures.

The primary aim of this study was to determine how often complications occur in children undergoing anesthesia outside the operating room for diagnostic and therapeutic procedures in our institution. Secondary aims were to describe the types and severity of those complications; identify risk factors; evaluate whether pre-anaesthetic consultation offers protection; assess how patients’ health status and comorbidities influence complication rates; and make recommendations to improve safety in pediatric NORA.

This article was previously posted to the Research Square preprint server in March 2026 [7].

## Materials and methods

Study design and setting

This prospective observational study was conducted over 10 months in the radiology and endoscopy departments of a university hospital. Ethical approval was obtained from the institutional review board, and informed consent was secured from parents or legal guardians.

Study population

All consecutive eligible patients meeting the inclusion criteria during the study period were included.

Sample size determination

The sample size was determined based on feasibility and the expected incidence of complications reported in previous studies of pediatric non-operating room anesthesia, estimated at approximately 10-20%. A minimum sample of 246 patients was calculated to estimate this proportion with a 95% confidence interval and a margin of error of 5%. To account for potential missing or incomplete data, the sample size was increased to 300 patients.

Children aged 0-12 years scheduled for elective or urgent diagnostic or therapeutic procedures under anesthesia or sedation outside the operating room (radiology or endoscopy units) were eligible. Only American Society of Anesthesiologists (ASA) physical status I-II patients were included. Exclusion criteria were emergency procedures, ASA ≥ III, known anesthetic allergy, refusal of consent, or incomplete data.

Anesthetic management

All patients were evaluated preoperatively by an anesthesiologist. When performed, preanesthetic consultations occurred within seven days of the procedure and included clinical assessment and airway evaluation. Fasting adhered to standard guidelines (6 h for solids, 4 h for breast milk, 2 h for clear fluids).

Anesthesia techniques included sevoflurane inhalation, intravenous propofol, or ketamine-midazolam combinations. In most cases, induction was performed using inhalational induction with sevoflurane (volatile induction and maintenance anesthesia (VIMA) technique), particularly in younger or uncooperative children. Airway management used a nasal cannula, face mask, or high-flow oxygen with spontaneous ventilation. Standard monitoring comprised continuous pulse oximetry, ECG, and non-invasive blood pressure every five minutes; capnography was added when available. Procedures were performed by specialty teams with an anesthesiologist present throughout.

Data collection

A standardized form recorded demographics, medical history, ASA status, procedure characteristics (type, duration, operator experience), anesthetic technique, airway management, and preanesthetic consultation. Outcomes included intra- and post-procedural complications, their severity, interventions, and the need for ICU admission.

Definitions

In this study, the term “critically ill” refers to patients presenting with acute clinical conditions, such as respiratory distress or altered consciousness at the time of the procedure, despite being classified as ASA II according to standard definitions.

Complications were defined as any intra- or post-anesthetic adverse event requiring intervention. Respiratory complications included desaturation (SpO₂ < 90%), laryngospasm, bronchospasm, apnea, and airway obstruction. Cardiovascular complications included bradycardia, hypotension, and arrhythmia. Other complications included vomiting, emergence agitation, seizures, and prolonged unconsciousness.

Statistical analysis

Data were analyzed using SPSS v26 (IBM Corp., Armonk, NY, US). Continuous variables were compared using the Student’s t-test, while categorical variables were analyzed using the chi-square test. Test statistics (t-values and χ² values) were reported alongside p-values. Variables with p < 0.20 were included in multivariable logistic regression. A p < 0.05 was considered statistically significant.

## Results

Study population and incidence of complications

During the study period, a total of 300 children who underwent sedation or anesthesia in the radiology and endoscopy departments were included in this prospective observational study. Among these patients, 26 (8.7%) experienced at least one complication during or immediately after the procedure, constituting our complications group. The remaining 274 (91.3%) patients had uneventful procedures and formed the no-complications group.

The overall incidence of complications was 8.7% (95% CI: 5.8-12.4%). The mean age of the study population was 3.5 ± 2.4 years, with a mean weight of 13.8 ± 6.1 kg. Male patients represented 57.7% (n = 173) of the cohort.

Patient demographics and clinical characteristics

Table 1 presents the demographic and clinical characteristics of patients in both groups. Patients who experienced complications were significantly younger (mean age: 2.8 ± 2.1 years vs. 3.6 ± 2.4 years, p = 0.042) and had lower mean weight (11.9 ± 5.3 kg vs. 14.0 ± 6.2 kg, p = 0.048). There was no significant difference in gender distribution between groups (male: 61.5% vs. 57.3%, p = 0.663).

**Table 1:** Demographic and clinical characteristics of the study population SD: standard deviation; URI: upper respiratory infection; ASA: American Society of Anesthesiologists * p < 0.05 statistically significant

Variable	Complications (n = 26)	No Complications (n = 274)	Test Statistic	p-value
Age (years), mean ± SD	2.8 ± 2.1	3.6 ± 2.4	t = 2.05	0.042*
Weight (kg), mean ± SD	11.9 ± 5.3	14.0 ± 6.2	t = 1.99	0.048*
Male gender, n (%)	16 (61.5%)	157 (57.3%)	χ² = 0.19	0.663
ASA I, n (%)	14 (53.8%)	177 (64.6%)	χ² = 1.28	0.256
ASA II, n (%)	12 (46.2%)	97 (35.4%)	χ² = 1.28	—
Neurological disease, n (%)	8 (30.8%)	62 (22.6%)	χ² = 0.90	0.344
Respiratory disease, n (%)	11 (42.3%)	85 (31.0%)	χ² = 1.48	0.223
Cardiac disease, n (%)	2 (7.7%)	8 (2.9%)	χ² = 1.82	0.177
Recent URI (<15 days), n (%)	3 (11.5%)	6 (2.2%)	χ² = 6.30	0.012*
CT scan, n (%)	12 (46.2%)	135 (49.3%)	χ² = 0.10	0.752
MRI, n (%)	9 (34.6%)	95 (34.7%)	χ² = 0.00	0.992
Bone marrow biopsy, n (%)	2 (7.7%)	24 (8.8%)	χ² = 0.03	0.853
Endoscopy, n (%)	3 (11.5%)	20 (7.3%)	χ² = 0.60	0.439
Duration (min), mean ± SD	15.8 ± 10.9	17.2 ± 11.3	t = 0.64	0.522

Patients with ASA II classification represented 46.2% of the complications group compared to 35.4% in the no-complications group, though this difference did not reach statistical significance (p = 0.256). A history of recent upper respiratory infection within 15 days was significantly more common in the complications group (11.5% vs. 2.2%, p = 0.012).

Anesthetic management

Table 2 summarizes the anesthetic techniques and agents used during the procedures. Sevoflurane was the most commonly used anesthetic agent (87.3%, n = 262), followed by propofol (8.3%, n = 25), and ketamine with midazolam combination (4.3%, n = 13).

**Table 2:** Anesthetic management characteristics IV: intravenous; ICU: intensive care unit * p < 0.05 statistically significant

Variable	Complications (n = 26)	No Complications (n = 274)	Test statistic	p-value
Sevoflurane, n (%)	21 (80.8%)	241 (88.0%)	χ² = 1.13	0.287
Propofol, n (%)	3 (11.5%)	22 (8.0%)	χ² = 0.39	0.532
Ketamine + Midazolam, n (%)	2 (7.7%)	11 (4.0%)	χ² = 0.88	0.347
High-flow face mask, n (%)	19 (73.1%)	211 (77.0%)	χ² = 0.21	0.644
Nasal cannula, n (%)	5 (19.2%)	50 (18.2%)	χ² = 0.01	0.905
Facial mask, n (%)	2 (7.7%)	13 (4.7%)	χ² = 0.49	0.481
IV line established, n (%)	7 (26.9%)	81 (29.6%)	χ² = 0.08	0.770
Preanesthetic consultation, n (%)	8 (30.8%)	152 (55.5%)	χ² = 6.12	0.013*
Specialist, n (%)	21 (80.8%)	228 (83.2%)	χ² = 0.10	0.747
Resident, n (%)	4 (15.4%)	38 (13.9%)	χ² = 0.04	0.831
Professor, n (%)	1 (3.8%)	8 (2.9%)	χ² = 0.07	0.789
Recovery on table, n (%)	19 (73.1%)	239 (87.2%)	χ² = 4.22	0.040*
Recovery room/ICU, n (%)	7 (26.9%)	35 (12.8%)	χ² = 4.22	—

Preanesthetic consultation within a few days before the NORA procedure was performed in only 30.8% of patients in the complications group compared to 55.5% in the no-complications group (p = 0.013), suggesting a protective effect. Recovery location differed significantly between groups, with more patients in the complications group requiring recovery room or ICU admission (26.9% vs. 12.8%, p = 0.040).

Types and severity of complications

Table 3 describes the types and frequencies of complications observed during NORA procedures. Respiratory complications were the most frequent (57.7%, n = 15), followed by emergence agitation (30.8%, n = 8), cardiovascular complications (11.5%, n = 3), and gastrointestinal complications (11.5%, n = 3).

**Table 3:** Types and distribution of complications Some patients experienced more than one complication.

Type of Complication	n	Percentage of Complications (%)	Incidence in Total Cohort (%)
Respiratory complications	15	57.7	5.0
Desaturation (SpO₂ < 90%)	11	42.3	3.7
Cough	1	3.8	0.3
Bronchospasm	3	11.5	1.0
Emergence agitation	8	30.8	2.7
Cardiovascular complications	3	11.5	1.0
Bradycardia	3	11.5	1.0
Gastrointestinal complications	3	11.5	1.0
Vomiting	3	11.5	1.0
Neurological complications	1	3.8	0.3
Convulsive emergence delirium	1	3.8	0.3
Total patients with complications	26	100	8.7

Desaturation (oxygen saturation below 90%) was the single most common complication, occurring in 11 patients (3.7% of the total cohort, 42.3% of complications). All desaturation episodes were successfully managed using non-invasive measures, including airway repositioning, increased oxygen supplementation, and, in some cases, temporary interruption of the procedure. No oropharyngeal airway (OPA) or nasopharyngeal airway (NPA) placement was required. Importantly, no patient experienced oxygen desaturation below 80%, and no endotracheal intubation was necessary.

Among the 26 complications, 20 (76.9%) were classified as minor, requiring minimal intervention, while 6 (23.1%) were major complications necessitating significant therapeutic interventions or unplanned transfer. One patient with convulsive emergence delirium required transfer to the intensive care unit for observation and eventually recovered without sequelae. No mortality was observed in this cohort.

Risk factors for complications

Univariable Analysis

Table 4 presents the results of univariable logistic regression analysis identifying potential risk factors for complications during NORA. Several variables showed significant associations with increased risk of complications in the univariable analysis.

**Table 4:** Univariable analysis of risk factors for complications OR: odds ratio; CI: confidence interval; URI: upper respiratory infection; ASA: American Society of Anesthesiologists; IV: intravenous * p < 0.05 is statistically significant

Variable	Complications (%)	No Complications (%)	OR	95% CI	Test statistic	p-value
Age < 2 years	46.2	28.5	2.159	0.975–4.783	χ² = 3.62	0.057
Weight < 10 kg	38.5	23.4	2.046	0.903–4.637	χ² = 2.98	0.085
Male gender	61.5	57.3	1.194	0.529–2.696	χ² = 0.19	0.663
ASA II	46.2	35.4	1.564	0.711–3.442	χ² = 1.24	0.265
Neurological disease	30.8	22.6	1.514	0.633–3.619	χ² = 0.87	0.350
Respiratory disease	42.3	31.0	1.634	0.736–3.628	χ² = 1.46	0.227
Cardiac disease	7.7	2.9	2.784	0.570–13.593	χ² = 1.62	0.203
Recent URI	11.5	2.2	5.821	1.377–24.600	χ² = 5.69	0.017*
Difficult intubation	7.7	1.8	4.481	0.830–24.202	χ² = 3.03	0.081
Current URI	7.7	1.5	5.563	0.986–31.396	χ² = 3.77	0.052
Altered consciousness	7.7	0.7	11.333	1.531–83.948	χ² = 5.69	0.017*
Respiratory distress	19.2	2.9	7.889	2.413–25.801	χ² = 13.1	<0.001*
Preanesthetic consultation	30.8	55.5	0.358	0.153–0.836	χ² = 5.60	0.018*
IV access	26.9	29.6	0.878	0.362–2.128	χ² = 0.08	0.771
Duration > 20 min	34.6	32.5	1.099	0.478–2.524	χ² = 0.05	0.824

The strongest predictors in univariable analysis were: altered consciousness at presentation (OR = 11.333; 95% CI: 1.531-83.948; p = 0.017), respiratory distress (OR = 7.889; 95% CI: 2.413-25.801; p < 0.001), and recent upper respiratory infection within 15 days (OR = 5.821; 95% CI: 1.377-24.600; p = 0.017).

Preanesthetic consultation performed a few days before the procedure was associated with a significantly reduced risk of complications (OR = 0.358; 95% CI: 0.153-0.836; p = 0.018), suggesting a protective effect.

Multivariable Analysis

Variables with p-values < 0.20 in the univariable analysis were entered into the multivariable logistic regression model. Table 5 shows the results of the multivariable analysis after adjusting for potential confounders.

**Table 5:** Multivariable logistic regression analysis for predictors of complications OR: odds ratio; CI: confidence interval; URI: upper respiratory infection * p < 0.05 statistically significant

Variable	Adjusted OR	95% CI	p-value
Respiratory distress	6.845	1.986-23.583	0.002*
Recent URI (<15 days)	4.921	1.042-23.230	0.044*
Altered consciousness	9.127	1.124-74.123	0.038*
Predicted difficult intubation	3.842	0.632-23.357	0.145
Preanesthetic consultation (protective)	0.389	0.154-0.982	0.046*

After adjusting for confounders, four variables remained independently associated with complications. The most significant predictor was respiratory distress at presentation (aOR = 6.845; 95% CI: 1.986-23.583; p = 0.002), followed by altered consciousness (aOR = 9.127; 95% CI: 1.124-74.123; p = 0.038), and recent upper respiratory infection (aOR = 4.921; 95% CI: 1.042-23.230; p = 0.044).

Preanesthetic consultation remained a significant protective factor after adjustment (aOR = 0.389; 95% CI: 0.154-0.982; p = 0.046), indicating that proper preanesthetic evaluation reduced the risk of complications by approximately 61%.

The multivariable model showed good discriminative ability (C-statistic = 0.782; 95% CI: 0.699-0.865) and adequate calibration (Hosmer-Lemeshow test: χ² = 6.824, p = 0.447).

Clinical status and patient condition

Analysis of patient clinical status revealed that critically ill children (defined as those with respiratory distress, altered consciousness, or ASA II classification with acute illness) had a significantly higher complication rate. Of the 26 patients with complications, 15 (57.7%) were classified as critically ill compared to 49 (17.9%) in the no-complications group (OR = 6.256; 95% CI: 2.723-14.372; p < 0.001).

Outcomes and recovery

All patients eventually recovered and were discharged in stable condition. The one patient requiring ICU admission had convulsive emergence delirium and was discharged from the ICU after 24 hours of observation without sequelae. No mortality was observed in this cohort.

## Discussion

This prospective observational study evaluated the incidence, nature, and predictors of complications during pediatric NORA and sedation procedures. Among 300 children managed in radiology and endoscopy units, the overall complication rate was 8.7% (95% CI 5.8-12.4%). The majority of adverse events were respiratory in nature, followed by emergence agitation and cardiovascular instability. Several clinical predictors were identified, including respiratory distress, altered consciousness, and recent upper respiratory infection, while pre-anesthetic consultation emerged as a significant protective factor. The observed 8.7% incidence aligns with recent data from low- and middle-income countries. Jarraya et al. reported a similar rate of 8.6% among 256 children in Tunisia [6], while Amin et al. [5] found a lower incidence of 3.2% in a larger multicentric cohort. In contrast, studies from high-resource settings, including the Pediatric Sedation Research Consortium [8-11], have reported complication rates between 5% and 7%. These findings confirm that pediatric NORA procedures carry a non-negligible risk of complications, particularly in radiology and endoscopy environments where airway management and logistical constraints are challenging [2,3].

Respiratory complications were the most frequent in our study, accounting for 57.7% of all adverse events (5% of cases), consistent with previous reports [5,12]. Similar trends were observed in Mongodi et al. [13], where 50% of adverse events were respiratory, predominantly airway obstruction and laryngospasm, and in Sirimontakan et al. [14], who reported a 3.9% incidence of respiratory complications. Large multicenter registries also highlight hypoxemia and airway compromise as the leading adverse events, with Cravero et al. reporting oxygen desaturation in 1.6% and laryngospasm in 0.043% of cases [10]. The predominance of respiratory events reflects both the anatomical and physiological vulnerability of children and the constraints inherent to non-operating room environments. Younger age and lower body weight were significantly associated with adverse outcomes, consistent with Ferrazzano et al. [12]. In our cohort, respiratory distress (aOR = 6.845, p = .002), altered consciousness (aOR = 9.127, p = .038), and recent upper respiratory infection (aOR = 4.921, p = .044) were independent predictors of complications, in agreement with Jarraya et al. [6] and Beach et al. [15].

Given the increased risk associated with upper respiratory infections (URIs), several guidelines recommend postponing elective procedures for approximately two weeks after symptom resolution in uncomplicated cases [16], and up to four weeks or more in severe infections [17,18]. Risk stratification tools such as the COLDS score further support individualized decision-making [19]. In our study, pre-anesthetic consultation significantly reduced the risk of complications by 61% (aOR = 0.389; p = 0.046), highlighting its critical role in identifying risk factors and optimizing patient management. This finding is consistent with previous studies emphasizing the importance of structured pre-procedure assessment and protocol standardization in improving NORA safety [6,20]. Regarding anesthetic techniques, sevoflurane was the most frequently used agent, yet no specific drug was independently associated with complications. This supports prior findings suggesting that patient-related and procedural factors outweigh pharmacological choice in determining outcomes [5,12].

In our study, “critically ill” patients, defined pragmatically as those presenting with acute physiological disturbances despite ASA II classification, had a significantly higher risk of complications (OR = 6.256; p < 0.001), consistent with Jarraya et al. [6]. While Beach et al. [15] did not identify acute illness alone as a major predictor, they demonstrated that higher ASA status and procedural urgency increase risk. Importantly, no mortality was observed in our cohort, likely reflecting effective monitoring and prompt intervention. These findings have important clinical implications: children with respiratory or neurological compromise should be managed in appropriately equipped settings with trained personnel and immediate access to airway support. Standardization of NORA practices, including systematic pre-anesthetic evaluation, use of checklists, and adherence to monitoring standards equivalent to the operating room, has been strongly advocated in the literature [2,5,20,21]. Furthermore, most complications in our study were managed with basic airway maneuvers and supportive measures, underscoring the importance of fundamental airway skills, structured training, and simulation-based education to ensure safety in these remote environments.

Limitations

This study has several limitations. First, its observational design precludes establishing causal relationships between risk factors and complications. Second, it was conducted in a single center, which may limit the external validity and generalizability of the findings to other settings with different patient populations and clinical practices. Although consecutive inclusion was applied, selection bias cannot be completely excluded. Information bias related to data recording and potential variability in reporting complications may also be present. In addition, heterogeneity in anesthetic techniques and operator experience may have introduced performance bias. The sample size, while sufficient for estimating overall complication rates, remains relatively modest compared with multicenter studies and may limit the detection of rare adverse events. Furthermore, long-term outcomes were not assessed, and subtle or delayed complications, such as neurobehavioral effects, may have been underestimated.

## Conclusions

This prospective analysis identified a complication rate of 8.7% in pediatric non-operating room anesthesia, predominantly respiratory in nature. The most powerful predictors of complications were respiratory distress, altered consciousness, and recent upper respiratory infection, whereas pre-anesthetic consultation significantly reduced risk. These results, in harmony with recent international evidence, highlight the critical value of systematic pre-anesthesia assessment for procedural preparedness and multidisciplinary coordination in improving safety for pediatric NORA patients.
